# Idiopathic High-Flow Priapism

**DOI:** 10.34172/aim.2023.88

**Published:** 2023-10-01

**Authors:** Mohamed Zouari, Emna Krichen, Nesrine Ben Saad, Najoua Kraiem, Wiem Rhaiem, Riadh Mhiri

**Affiliations:** ^1^Department of Pediatric Surgery, Hedi-Chaker Hospital 3029 Sfax, Tunisia; ^2^Faculty of Medicine, University of Sfax, Sfax, Tunisia

 A previously healthy 5-year-old boy presented to the emergency unit with persistent erection for 6 hours. The patient had undergone circumcision at the age of 2 years with no incidents. He felt no pain and was not on any medication. The mother reported intermittent priapism during the last week. The boy had no history of recent perineal or penile trauma. According to the mother, there was no change in the patient’s behavior or voiding disorders.

**Figure 1:**
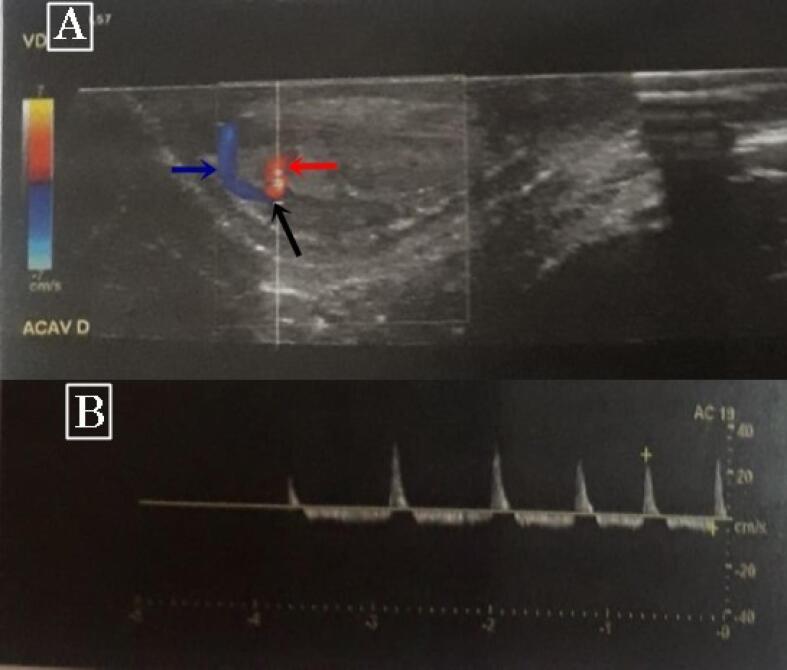
Color Doppler Ultrasonography. **(A)** Red, blue and black arrows show arterial blood flow, venous blood flow, and arteriolar-sinusoidal fistula, respectively. **(B)** High blood flow rate at the level of the fistula

 Physical examination showed an erect, normally circumcised, non-tender penis. The corpora cavernosa was rigid and the glans was soft. The laboratory work-up, including a complete blood count and a cytobacteriological examination of the urine, was normal. Penile color Doppler ultrasound revealed an arteriolar-sinusoidal fistula at the base of the left corpus cavernosum with a high flow rate of 34 mL/s at the level of the fistula (Figure 1). Based on these findings, we opted for a conservative treatment consisting of analgesics with clinical observation. Two days after hospital admission, the priapism resolved spontaneously. The child has been followed as an outpatient for 3 years, and has not experienced any recurrence to the present time.

 Priapism is a non-sexually related persistent erection lasting over 4 hours.^1^ Priapism is uncommon in the pediatric population, occurring in 0.3 to 1.5 per 100 000 children per year.^2^ The management of priapism is usually challenging. This condition can be serious leading to structural damage to the penis with irreversible compromise of the erectile function. Priapism has been divided into 3 main categories based on physiopathological grounds: ischemic, non-ischemic, and stuttering.^3^ Ischemic priapism is the most common entity accounting for 95% of all episodes. It is an emergency that must be treated without delay. The treatment of ischemic priapism is based on many strategies. The first one includes a combination of blood aspiration and intracavernous α-agonist therapy. Second-line therapy is based on surgical shunts. Penile prosthesis implantation is the last therapeutic strategy.^4^

 Stuttering priapism is described as multiple episodes of painful and unwanted erections, which are often self-limiting. This form of priapism occurs commonly in patients with sickle cell disease and can progress to a more serious form of ischemic priapism.^5^

 High-flow priapism also called non-ischemic priapism is a partial erection due to lack of regulation of the cavernous arterial inflow. This condition is usually caused by a penile trauma leading to laceration of cavernous arterioles and the creation of an arteriolar-sinusoidal fistula. This results in a continuous arterial blood flow to the penis.^6^ Non-ischemic priapism may be also idiopathic due to congenital arterial malformations.^7^ High-flow priapism does not cause ischemia and does not lead to a painful erection in most cases. For these reasons, this condition is not considered as an emergency. Moreover, in most cases of high-flow priapism, the natural course is towards spontaneous resolution.^6^

 Patients with high-flow priapism usually present with non-tender, partially tumescent corpora cavernosa. In such cases, Doppler ultrasound examination of the penis is the gold standard for the diagnosis. It can assess intracorporeal arterial blood flow in real time. In patients with non-ischemic priapism, Doppler ultrasonography of the penis shows normal or increased velocity of blood flow in the corporal arteries.^8^ It can also reveal an arteriolosinusoidal fistula, as in our patient’s case.

 The initial treatment strategy for non-ischemic priapism is quite different from that for ischemic or stuttering priapism. Most authors recommend conservative management as the first-line treatment for this type of priapism.^6^ Therefore, over 70% of all episodes of high flow priapism resolve spontaneously by clinical observation alone.^1^ While aspiration is not recommended, surgery should be considered in refractory cases.^1,6,9^

 Priapism is an uncommon childhood condition. The recognition of ischemic and non-ischemic forms is crucial, implying completely different diagnoses and management strategies.
